# Facilitators and strategies to implement clinical pharmacy services in a metropolis in Northeast Brazil: a qualitative approach

**DOI:** 10.1186/s12913-018-3403-4

**Published:** 2018-08-13

**Authors:** Sheila Feitosa Ramos, Genival Araujo dos Santos Júnior, André Mascarenhas Pereira, Aline Santana Dosea, Kérilin Stancine Santos Rocha, Déborah Mônica Machado Pimentel, Divaldo Pereira de Lyra-Jr

**Affiliations:** 1 Laboratory of Teaching and Research in Social Pharmacy (LEPFS), Department of Pharmacy, Federal University of Sergipe, Cidade Universitária “Prof. José Aloísio Campos”, Jardim Rosa Elze, São Cristóvão, Zip code: 49100-000 Brazil; 2 Department of Medicine, Federal University of Sergipe, Cidade Universitária “Prof. José Aloísio Campos”, Jardim Rosa Elze, São Cristóvão, Zip code: 49100-000 Brazil

**Keywords:** Facilitators, Implementation strategies, Implementation, Clinical pharmacy services, Health systems

## Abstract

**Background:**

Clinical Pharmacy Services (CPS) are a reality in many health systems around the world. However, there are few studies that discuss the facilitators and the strategies to implement CPS in healthcare systems. In this way, the objective of this study was to identify the facilitators and strategies involved in the CPS implementation process in some public health units in a metropolis in the Northeast Brazil.

**Methods:**

A qualitative study was carried out with health-system pharmacists and managers who experienced the implementation of CPS. Therefore, focus groups were conducted with pharmacists, and the interviews with the managers. The discussions were carried out through semi-structured scripts and were recorded in audio and videos, after the signature of the consent form. The recordings were transcribed and analyzed independently through content analysis, followed by consensus meetings between researchers.

**Results:**

Two focus groups were conducted, with an average of seven pharmacists per group, and five interviews with local health managers. Participants reported 39 facilitators who were related to the categories: local healthcare network, healthcare team, pharmacists and implementation process of the CPS. And 21 strategies attributed to the following categories: local healthcare network, pharmacists and implementation process of the CPS.

**Conclusions:**

This study identified facilitators and strategies of the implementation of CPS. Most of the positive experiences were related to the clinical skills and proactive attitudes of pharmacists. These findings may support pharmacists and health managers to implement CPS in health systems.

**Electronic supplementary material:**

The online version of this article (10.1186/s12913-018-3403-4) contains supplementary material, which is available to authorized users.

## Background

Over the past years, the increase of drug-related morbidity and mortality has caused losses in different health systems in countries such as Canada [1], the United States of America [2], Spain [3] and Brazil [4]. For this reason, the implementation of clinical pharmacy services (CPS) in health systems has been considered a social need worldwide [5–9]. CPS is a professional service provided by pharmacists, who use their skills and knowledge to improve patients’ pharmacotherapy [10].

The literature shows that CPS have expanded [11, 12] and have been carried out in different setting, as community pharmacy [13], ambulatory care facilities [14] and hospitals [15], showing a clinical [16], economic [17] and humanistic [18] positive impact. In Brazil, CPS have been expanding due to the legal support awarded by regulatory agencies [19, 20], Brazilian Federal Pharmacy Council [21, 22] and by initiatives of the Brazilian Government [23]. These actions encourage pharmacists to assume clinical responsibilities and provide CPS within the Brazilian health system.

In this scenario, it is highlighted the implementation science, since it is responsible for investigating and understanding the multiple factors that influence the implementation processes [24]. The literature has emphasized that the complexity of the CPS implementation process, especially in health systems [25, 26]. This complexity is characterized by interrelated stages, and influenced by multiple factors [27, 28].

Although identifying and understanding implementation factors may represent a key process in the implementation of CPS, there is a lack of studies focusing on facilitators and strategies for the CPS implementation process in healthcare systems [7, 29, 30]. Thus, the aim of this study was to identify the facilitators and strategies involved in the CPS implementation process in some public health units in a metropolis in northeast Brazil.

## Methods

### Study context

The Brazilian health system is a universal system that guarantees health services and drugs to all Brazilian citizens at the primary, secondary and tertiary care [31, 32]. In this system, the pharmacists have focused their practice on logistics activities, regardless of their workplace, because Brazilian Pharmacy undergraduate courses did not prioritize the development of clinical knowledge, skills and attitudes. However, the number of strategies to increase this pharmacists’ performance in clinical activities has been growing [33]. Thus, the present study was developed while the Brazilian ministry of health was implementing CPS in some public health units in a metropolis in Northeast Brazil. In this local healthcare network, there were no CPS.

The Brazilian ministry of health develop the CPS implementation process in four steps, from July 2015 to March 2016. In the first step, a collaborative partnership was formed between the Brazilian ministry of health and local health managers to ensure provision of structural conditions (physical and human resources) for the implementation of CPS. The second stage corresponded to the hiring of a team of specialists (supporters) with experience in the implementation of CPS in the Brazilian health system. The third stage related to the implementation of CPS through clinical training of 42 health-system pharmacists based on the Pharmacotherapy Workup [34]. Finally, the last step corresponded to the accreditation by the Brazilian ministry of health to 23 pharmacists who implemented CPS in their workplaces.

### Study design

A qualitative study was performed through two focus groups and five semi-structured interviews with pharmacists and local health managers involved in the CPS implementation process in order to explore their perceptions. The focus groups and interviews were conducted in April and August 2016, respectively.

This study used the recommendations proposed by Consolidated Criteria for Reporting Qualitative Research (COREQ) [35]. Additionally, the Brazilian Committee of Ethics in Research approved this study under registration CAAE number 35440114.0.0000.0008.

### Participants

This study was conducted with 13 health-system pharmacists and five local health managers that were involved in the CPS implementation. The pharmacists who consented to participate were those who agreed to implement CPS in their respective workplaces. In order to get a comprehensive understanding of the facilitators that influenced the CPS implementation process, the health-system pharmacists were divided into two groups: (i) seven accredited pharmacists, who fulfilled all steps of the process and implemented CPS in their workplaces; and (ii) five non-accredited pharmacists, who did not complete all steps of the process and did not fulfill implement CPS in their workplaces. The local health managers were those who held important positions in the local healthcare network (directors, supervisors, and coordinators) during the implementation of CPS.

### Topic guide of focus groups and interviews

The topic guide was formulated from a brainstorming meeting with the authors and has open questions that had the objective to investigating barriers, facilitators and strategies to implement CPS (Additional file 1). However, the quantity and diversity of the collected data and the need for a comprehensive understanding of the CPS implementation process (which is complex and multifactorial) led us to divide the data into two articles. The present article discusses the facilitator and strategies to implement CPS. The barriers are discussed elsewhere [36].

### Structure of focus groups and interviews

Focal groups and interviews were conducted with health-system pharmacists, and local health managers, respectively, and independently, as recommended in the literature [35]. Moderators (ASD and GASJ) with prior experience in conducting qualitative studies carried out these interviews. Moderators were advised to stop collecting data when new perspectives stopped appearing (data saturation).

At the beginning of this process, the moderators explained the research objectives and the participants agreed to collaborate by signing an informed consent authorizing the researchers to use their audio and video recordings. Focus group interviews were conducted in secluded environments, conducive to ensuring confidentiality and freedom to express, so that participants could share their perceptions. Individual times were scheduled for the interviews with the local health managers.

### Data collection and analysis

All the discussions generated were audio−/video-taped and all their contents were transcribed verbatim in full. Subsequently, transcripts were independently analyzed by a researcher who was involved in the data collection (GASJ) and two external researchers (AMP and SRF) based on the content analysis proposed by Bardin [37].

The data collected were coded, categorized, and organized during consensus meetings between the researchers. In these meetings, each researcher commented on the decision of the other researcher until they reached a consensus on the interpretation of the data. In order to increase methodological accuracy, at the end of this stage, two senior researchers (DPLJ and DMMP) reviewed all the data collected.

This method was carried out in multiple steps and with several different researchers and evaluators (independently or in consensus meetings) to achieve the reliability and validity of the data. Finally, we emphasize that the triangulation was performed throughout the step-by-step execution of the methods. Thus, we used different data collection techniques (focus groups and interviews), several evaluators (internal and external researchers) to interpret the transcripts, and two methods for data analysis (consensus meetings and independent evaluations). These methods used of triangulation in qualitative research, based on concept proposed by Carter et al. [38].

### Concepts adopted

Facilitators are the factors or the determinants of practice that influence the implementation effort [39]. In this study, we considered as facilitators, the factors listed by the pharmacists and local health managers who made the CPS implementation process easier. While strategies are a systematic intervention process to adopt and integrate evidence-based health innovations into usual care [40], in this study, we consider the interventions proposed by pharmacists and local healthcare managers to implement CPS as strategies.

## Results

Two focus groups were conducted, one with seven accredited pharmacists and the other one with five non-accredited pharmacists. The interviews with key informants were carried out with five local health managers. The local health managers were two pharmacists, two physicians, and one occupational therapist.

The focus group and interviews discussions generated 140-min and 100-min recordings, respectively. Participants reported 39 facilitators and 21 strategies related to CPS implementation. To aid understanding, the facilitators were related to four categories: local healthcare networks, healthcare teams, pharmacists, and implementation process of CPS. The strategies were attributed to three categories: local healthcare networks, pharmacists, and the implementation process of CPS. Tables 1 and 2 show all facilitators and strategies for implementing CPS, respectively.

**Table 1:** Facilitators for implementation of CPS, reported by pharmacists and local health managers

Categories	Accredited pharmacists	Non-accredited pharmacists	Local health managers
Local healthcare network	-Support of some local health managers -Organizational structure of the local healthcare network -Adequate physical structure of healthcare units -Easy access of pharmacists to patients -Support of pharmacy staff	-Support of local health managers -Clinical profile of healthcare units -Physical proximity between the pharmacist’s workplace and the health units -High demand of patients in health units	-Commitment of management to ensure the sustainability of CPS -Collaborative agreement between managers and the Ministry of Health to implement CPS -Local healthcare network and local pharmaceutical services organized -Large number of pharmacists in local healthcare network -Outsourcing of the main activities performed by local pharmaceutical services
Health team	- Collaboration of health team	- Collaboration of health team	-Support of health team
Pharmacists	-Proactivity and commitment -Motivation with positive preliminary results of the implementation of CPS -Pre-existing clinical profile -Previous knowledge -Process work in collaboration with health team	-Communication with health team -Access to the patient’s medical record -Pre-existing patient-pharmacist interaction	-Availability and commitment -Possibility of pharmacists working in a different setting -Pharmacists who graduated recently
Implementation process of the CPS	-Technical support during CPS implementation -Theoretical-practical training offered to pharmacists -Systematization of the work process of clinical activities through medical records	Not related	-Expertise of supporters in implementing CPS -Mentoring activities carried out by supporters to implement CPS -Implementation process of CPS had been started in collaboration with the Ministry of Health -Challenge pharmacists to implement CPS -Non-authoritative participation of the pharmacists -Previous successful experience of the Ministry of Health in the implementation of CPS in another city -Implementation process of CPS adapted in the local region

**Table 2:** Strategies for implementation of CPS, reported by pharmacists

Categories	Accredited pharmacists	Non-accredited pharmacists
Local healthcare network	- Participating actively in the implementation process of CPS - Ensuring minimum structural conditions - Ensuring presence of pharmacy interns	- Participating actively in the implementation process of CPS - Ensuring minimum structural conditions
Pharmacists	- Performing the work process with attentive and careful attitude towards the patient - Engaging in the CPS implementation process - Acquiring the necessary support materials for CPS with one’s own resources - Getting patients by active search - Training pharmacy staff to perform logistic activities - Sensitizing health team on CPS - Sensitizing patients on CPS	- Delegating logistic activities to pharmacy staff - Delegating patient recruitment to health team - Reconciling logistic and clinical activities - Disclosing the CPS - Sensitizing the health team - Getting patients for CPS
Implementation process of the CPS	- Adapting the CPS according to the workplace and patients’ health needs - Selecting the pharmacists who will implement the CPS according to clinical profile - Extending the time for CPS implementation	–

### Facilitators

#### Facilitators related to the local healthcare networks

In the focus group and interviews, it was reported that facilitators related to the local healthcare network and characteristics of the local management model. All participants of this study pointed out the commitment of the local management as a facilitator, which showed that accessibility and responsiveness in providing material resources were necessary in the implementation of CPS.

“The room was acclimatized, the computer was placed for me to attend [the patients]; after a request to the local management, it was quiet to perform patient care. I can attend to them without difficulty” (Accredited Pharmacist D).

Regarding the structure of the local healthcare network, the organization of services, health facilities, and local pharmaceutical services was reported to be a facilitator. Some units presented infrastructure as a facilitator, with adequate and comfortable physical space for pharmaceutical care (private acclimatized room), computer equipment, and clinical devices (tensiometer, glucometer, etc.).

“We always had a room available, and we received material from the local health management” (Accredited Pharmacist G).

Some reported facilitators were related to certain health units where pharmacists worked, characterized by high demand and easy access to patients. These attributes facilitated the sensitization and capitation of patients to CPS. In addition, it was mentioned that the service profile of some units (for example, mental health services) favored the clinical performance of pharmacists since these institutions offer integral care and interdisciplinary assistance, focusing on patients, families, and the community.

“I think the great facilitator is the high demand of patients and the very close contact we have with them. Both during care, when we do the drug dispensing, or even when talking to them” (Non-accredited Pharmacist E).

The pharmacists reported the support of pharmacy staff in the execution of logistic activities. These activities were delegated to the pharmacy staff. In this way, the pharmacists managed to organize their work process in order to dedicate more time to the CPS.

“Today, I can share with them [pharmacy staff] some routines that were not previously divided as they were focused on me. I gave them more autonomy” (Accredited Pharmacist D).

#### Facilitators related to the healthcare team

Focus group and interviews pointed collaboration and good communication among healthcare team members as an indispensable factor for the implementation of CPS. According to the participants of this study, the relationship of pharmacists with the healthcare team facilitated the referral of patients to CPS, improved the communication with other professionals, and increased the recognition of pharmacists within the healthcare team.

“They [healthcare team] were satisfied; they gave feedback and referred patients. This was a positive point to show the pharmacist's work to the healthcare team, especially when the physician reacted very well and they interacted a lot*”* (Accredited Pharmacist H).

#### Facilitators related to pharmacists

Accredited pharmacists reported facilitators related to their attitudes, as willpower, commitment, and proactivity to learn and perform clinical activities. The motivation generated by the positive results of CPS boosted the progress of these services. Managers also reinforced the availability and intellectual/personal commitment of pharmacists to obtain knowledge that would help the implementation of CPS.

“A great facilitator, in the first place, was my willpower [...] to want to do, to want to learn, despite the difficulties; I think the proactivity of each one was fundamental for this*”* (Accredited Pharmacy D).

The clinical profile was cited as a facilitator. We defined this profile as a set of intrinsic characteristics of a professional who is motivated and had an affinity for clinical activities, and previous knowledge, skills and attitudes. Some pharmacists reported having a previously created clinical profile to act directly with the patient, which was stimulated by the CPS implementation process. In addition, lifelong knowledge has helped pharmacists in clinical practice and patient management.

“I want to be with the patient [...] not only as a pharmacist, but […] like a human who wants to make a difference in the patient’ life, since the time they are diagnosed until the moment they know that it is possible to live with the disease and have a good quality of life” (Accredited Pharmacist G).

The pharmacists’ work process, integrating collaborative practices with other members of the healthcare team, in a different setting was pointed out as an important facilitator. In addition, access to medical records, previous pharmacist-patient interactions.

*“*I have full access to the patients' medical records, and physicians are accessible. With RT [reference technicians] we can talk about patients, like any healthcare team*”* (Non-accredited Pharmacist C).

#### Facilitators related to the implementation process of CPS

Participants reported that assistance provided by supporters during the implementation of CPS was an essential facilitator, both in theoretical and practical training, as mentoring activities during pharmaceutical care. The local health manager also recognized the expertise and contributions of supporters in CPS implementation. In addition, the systematization of the pharmacists’ work process through documentation in medical records helped assimilate and incorporate clinical activities into the work routine of the pharmacists.

“The biggest facilitator for me was the existence of supporters, without them the provision of CPS did not work, [...] to have a mentor able to tell you: ‘See, this will work, this will not work. Go that way, because this is the best way you can go’” (Accredited Pharmacist C).

The fact that the implementation process was initiated by the Brazilian ministry of health was mentioned as a facilitator. This brought confidence in the participants because of the previous positive experiences and they still felt challenged to implement CPS in their workplace.

“I think being challenged to do something different was one of the things that boosted [pharmacists] the most” (Local Health Manager D).

Non-accredited pharmacists, on the other hand, did not report facilitators related to CPS implementation process.

### Strategies to implement CPS

#### Strategies related to the local healthcare network

Some strategies were ensuring minimal physical infrastructure for services and more active participation of local health managers on the implementation process. In addition, accredited pharmacists emphasized the strategy to ensure the presence of pharmacy interns in the CPS implementation process.

“I do not think [local health management] giving us a glucometer in December is a support. Support is to be together, to attend.” (Non-accredited Pharmacist C).

#### Strategies related to pharmacists

Pharmacists proposed strategies they themselves should develop to facilitate the implementation process. Accredited pharmacists reported proactivity and humanized posture during clinical activities and recruited patients through an active search. The non-accredited pharmacists suggested delegating some activities to the pharmacy staff and delegating the recruitment of patients to the healthcare team.

*“*The best strategy is your work with patients [...] the way you work, how much you make a difference in their treatment [...]. It is the pharmacist in front of the patients, really looking at them, to listen. I realized that it is crucial that you listen to your patients” (Accredited Pharmacist G).

*“*I bought all basic materials [sphygmomanometer, stethoscope and lab coat] [...] because I think it is important for the achievement and success of the service*”* (Accredited Pharmacist E).

Several strategies were cited that aimed at strengthening relations between pharmacists, the healthcare team, and the patients. Thus, it was proposed that the pharmacy staff should be able to collaborate in logistic activities by sensitizing the healthcare team and patients, in addition to disseminating implemented CPS through lectures, waiting area approach, home visits, and shared care.

Non-accredited pharmacists pointed out the importance of strategies that would help reconcile the time between the logistic and clinical activities and how to delegate logistic activities so that new activities required for CPS implementation process were incorporated.

*“*I used shared care with nurses and physicians as a strategy, because in this way they could understand the process, the service, and what could be the result [...] they saw my way of intervening in care and then I created a bond for the next consultation.” (Accredited Pharmacist B).

#### Strategies related to the CPS implementation process

Some accredited pharmacists suggested some strategies that could be adopted in future implementation processes in local healthcare networks. According to them, it would be interesting to adapt the CPS according to social demands where the service will be offered and select the pharmacists according to their “clinical profiles”. According to the proposal, this could ensure better medication adherence and reduce the dropout rate during the process.

“There was a lack of selection on the professionals’ profile [...] there were many professionals who actually started doing this because they were involved, but they had no “clinical profile”, no capacity, or no help, either they had no infrastructure or, due to any other difficulty, they gave up the project and this was discouraging for other professionals too” (Accredited Pharmacist C).

## Discussion

The focus groups and interviews brought reflections to the implementation of CPS in certain public health units of a city in northeast Brazil. The accredited pharmacists showed themselves as protagonists in the situation, proposing facilitators inherent to the own attitudes. On the other hand, non-accredited pharmacists held a secondary role in which they attributed the protagonism to other people, such as the health team.

Tsoi et al. [41] explored factors related to motivation in clinical practice, and according to the authors, autonomy, personal desire, and pleasure in learning are related to motivation. These factors are considered essential facilitators in the professional development process and implementation services. While the lack of motivation is a state of passive behavior, in which pharmacists cannot achieve the expected results. In this way, the motivation can be influenced by factors of their own behavior or external factors and should be encouraged in the implementation process.

Local healthcare managers focused on factors related to the organizational structure of the municipal healthcare network. However, by the influence of their political positions, they may have overestimated some facilitators, such as emphasizing the adequate number of pharmacists available in the local healthcare networks. Political discourse can often be influenced according to the manager’s interest and/or the power of that position can be conveniently applied to emphasize some specific item or detail [42–44]. Therefore, it is necessary to pay attention to the influence political office has on managers’ speeches.

### Facilitators

#### Facilitators related to the local healthcare network

Similar to this study, several studies have shown that factors related to the commitment of healthcare managers, such as the availability of time and material resources, may contribute to an increase in the satisfaction of pharmacists, as well as encourage their autonomy and initiative to perform clinical activities [45–47]. Tapia-Conyer et al. [48] identified political support to be one of the key facilitators for the implementation of services. For these authors, when there is political support, innovative models are likely to be implemented more fully and more quickly. Thus, the involvement of managers in practice should be encouraged in order to ensure the resources needed for the successful implementation of CPS.

Regarding the physical structure, according to the model proposed by Donabendian [49], the implementation of health services, including CPS, can be carried out through three perspectives: structure (physical and human resources), processes (denotes what is actually done in giving and receiving care), and outcome (changes in the health status attributed to the interventions performed). According to this model, improvements in structure reflect improvements in the processes, and consequently, in patient outcomes [49, 50]. Therefore, it is fundamental to plan the structure of health services to be provided, as these can directly impact the quality of care given to the patient.

The clinical profile of care of certain health units at Brazil’s Mental Health Services (CAPS) was a facilitator. CAPS promote public comprehensive care for people with severe and persistent mental disorders in Brazil in a humane and integral way, holding a holistic view of the patient [51]. Silva and Lima [52], in a structural analysis performed at CAPS, identified a good functioning structure with individual treatment rooms, living space for the patient and professional interaction, and accessible medical records. Such factors make these centers conducive to the flow of patients requiring integrated services, including CPS [53, 54]. Due to the demand for integrated services, the healthcare team can be sensitized to the clinical performance of pharmacists with patients, families, and the community.

The presence of trained teams with defined and organized practices improves the work process of pharmacists, facilitating the implementation and ensuring quality in the services provided [55, 56]. The pharmacy technicians can assist pharmacists with their functions by performing drug-related managerial and administrative activities. The literature reveals that there is growing interest in using technicians as support for pharmacists to have more time to invest in activities centered on patient care [57–59]. As a result, it is necessary to invest in the qualifications of these professionals so that they realize safe practices. In Brazil, there is still no regulation for the role of pharmacy technicians.

#### Facilitators related to healthcare team

All participants of this study reported the collaboration with the health team as a facilitator. The literature has discussed the growing role of pharmacists as members of the healthcare team in primary care, contributing and acting directly on patient care [6, 60, 61]. Fazel et al. [62] performed a systematic review with meta-analysis to assess the effects of pharmacists’ interventions with the healthcare care team on patients with diabetes. The authors observed improvement in clinical outcomes with significant reduction in glycated hemoglobin, systolic blood pressure, and low-density lipoprotein cholesterol. Troung et al. [63] evaluated the satisfaction of healthcare professionals, most of them physicians, about the perceived impact of CPS in the management of diseases in primary care. They reported high satisfaction and improvement of patients with diabetes, hypertension, and pain management. Therefore, interdisciplinary practices should be encouraged as they may raise awareness of the role of the pharmacist in the health care team, as well as the importance of CPS implementation.

#### Facilitators related to pharmacists

The facilitators inherent to the pharmacists in the present study were related to knowledge, skills, and attitudes. These elements form the basis of clinical pharmacists’ competencies and directly reflect on patient care [64]. The clinical profile reported by pharmacists was in fact individual factors, such as willpower, motivation, knowledge, and proactivity, differ in each individual and are shaped by beliefs, education, training, and personal experiences of pharmacists, considered essential for service implementation [56, 65]. Patterson et al. [66] revealed some individual factors that can contribute to CPS provision, such as proactivity to create knowledge and to apply it in overcoming challenges, positive self-esteem, versatility, availability, and intellectual and personal commitment. Such factors can help the pharmacist become proficient, interact better with the healthcare team and patients, and perform better at work.

A point highlighted in the present study was the motivation generated by the positive results. The positive results (clinical, humanistic, and economic) generated by CPS can sensitize managers, the healthcare team, and the pharmacists by motivating them in the implementation process [67]. Motivation is a dynamic process that can be modified at any time depending on professional satisfaction [68]. Thus, motivation may be related to the way pharmacists deal with external circumstances and with factors they cannot control.

Another topic addressed in this study was the knowledge of pharmacists related to previous experiences with CPS. Alcântara et al. [9] investigated the perceptions of a group of pharmacists and other professionals about the implantation of the clinical pharmacy in a high complexity public hospital. Most emphasized that the clinical pharmacy experience of some pharmacists at the institution was one of the key facilitators to implement the CPS. In this way, pharmacists who have gained knowledge of past experiences may feel more prepared to implement CPS.

In relation to the work process, some pharmacists were included in healthcare teams composed of professionals from different areas of knowledge, who acted in an integrated way, sharing experiences with patients, families, and the community [69]. In the study by Reis et al. [70], the interdisciplinary characteristic of the healthcare team, combined with the fact that some pharmacists were included in the healthcare team before the implementation of the service, increased the team’s awareness of the importance of CPS that would be developed. In addition, the working process of pharmacists in contact with interdisciplinary groups may provide more learning opportunities [71]. In this sense, the implementation of CPS can be favored in places where there is an integrated interdisciplinary team.

Access to patients’ clinical information was considered a facilitator. The literature shows that one of the major limitations for pharmacists to provide care to patients in primary care is the lack of access to patients’ clinical information, such as medical records [72–74]. Thus, access to this information may facilitate the work process of pharmacists, as well as the implementation of CPS.

#### Facilitators related to the CPS implementation process

Pharmacists were assisted by specialists who trained them and offered mentoring activities in order to obtain the best performance in providing CPS. In studies on pharmacists receiving training, advantages in the provision of services, improved professional practice, and feeling more confident in patient care, were recognized [75–77]. Mentoring works as a dynamic and reciprocal relationship between a specialist with experience and expertise (mentor) and a beginner (the mentee), in order to guide and share experiences, is important to enhance the mentee’s professional development [78, 79]. In the health field, mentoring tools help practitioners improve patient outcomes as well as develop specific skills and behaviors [80–82]. Thus, mentoring may increase the impact of positive actions and contribute to the transition of a new leadership profile of pharmacists.

In this study, the systematization of the new work process through medical records was reported as a facilitator. A roadmap for advancing implementation of services in primary care described by Blanchard et al. [83] demonstrated that one of the main components necessary for the implementation of healthcare services was the systematization of the work process. In this way, it is essential to ensure that the work process is capable of being taught, is understandable, reproducible, and achievable. The authors also showed that it is important to follow a philosophy of practice as it guides a pharmacist’s behavior to be ethically appropriate, clinically accurate, and legal. Thus, it is necessary for processes to be well articulated to ensure that a common language is established.

In addition, according to the implementation process, the fact that the project started with the support of the Ministry of Health was considered an indispensable facilitator. Penm et al. [5] investigated factors that influenced the implementation of CPS from the point of view of pharmacists; it was observed that most reported that government support was crucial for the promotion of pharmacy services. Some studies show that it is in the interest of the government to implement these services, since they can reduce costs and improve the access of population to health [84, 85]. Therefore, support from government agencies may help accelerate the implementation of CPS.

In this study, voluntary participation of the pharmacists in the process of implementation of CPS was placed as positive. In a review, Luetsch et al. [86] explored pharmacists’ attitudes and attributes in relation to the implementation of cognitive services or role extension. For the authors, flexible and non-imposing attitudes, which make the pharmacists feel that they have control over their actions, positively influenced this behavior. Thus, letting pharmacists choose to participate in the service can positively influence their behavior.

### Strategies

#### Strategies related to the local healthcare network

Pharmacists attributed guarantee of minimum physical structure and effective participation of local health managers as local healthcare network strategies. Similarly, Minard et al. [87] explored the pharmacists’ perceptions of barriers and facilitators in the Implementation of Clinical Pharmacy Key Performance Indicators. Support before the implementation process was indicated as a facilitator. This support included the assistance of managers, technical support (physical structure), guidelines on how to participate in clinical activities, and inclusion of these clinical activities in working hours. Therefore, health authorities should ensure sufficient physical and human resources to collaborate with pharmacists in administrative and clinical tasks.

Another strategy reported to support pharmacists’ working process was the collaboration of interns. The main objectives of interns in pharmacies are defined between the training institution and the university. Overall, assignments are designed to cover key areas of pharmaceutical competence on-site training, providing support to pharmacists working process [88, 89]. Thus, investment in recruiting interns may support pharmacists and facilitate the implementation process.

#### Strategies attributed to pharmacists

One of the strategies mentioned by accredited pharmacists was the proactivity in the implementation of CPS, the engagement in the process in an attentive and humane manner, recognizing the patients’ needs and seeking to assist them. With these attitudes, pharmacists can impact the quality of service provided, recruit patients more easily, and stimulate the building of a relationship of trust between the patient and the pharmacist [90–92]. On the other hand, some non-accredited pharmacists pointed out strategies that reflected their passive attitudes in the implementation process, transferring some of their attributions to other actors, such as the health and pharmacy teams [30, 93]. This passive attitude may be related to the fact that pharmacists still feel insecure about their role and these issues can affect the delivery of CPS.

The training of pharmacy staff was mentioned as a strategy to support the provision of CPS. Similarly, Mackeigan et al. [94] performed a qualitative study to describe strategies used by community pharmacies to implement a medication review service. The strategies were related to human resource management, such as staff training, to give support in administration. Such strategies can reduce the technical activities of pharmacists, freeing them to perform cognitive activities related to patient care.

Raising awareness among the health team through the disclosure of CPS has been reported as an essential strategy. To sensitize the healthcare team, the disclosure of the impact of pharmaceutical services through media, posters, and lectures was used as a strategy. Such marketing strategies have been successful in other similar studies [95, 96]. According to Ung, Harnett and Hu [97], the healthcare team’s ability to understand the role of pharmacists is important in order to avoid possible barriers. Finally, the dissemination of CPS can be a valid strategy to give visibility to pharmacists both for the healthcare team and the community, as well as to improve health team adherence.

The non-accredited pharmacists mentioned that a valid strategy for the implementation of CPS would be a reconciliation between logistics and clinical activities. Some professionals may see the insertion of new activities in their routine as increasing workload [87]. Similarly, some studies show that one of the main difficulties in this process is to reconcile clinical and logistic activities. [84, 87, 98]. For Garcia-Cardenas et al. [25], several factors can contribute to the lack of time, such as lack of priority, commitment, and poor distribution of activities. In this way, it is fundamental to understand the specific causes that contribute to the lack of time, so that measures are developed to remedy this problem.

#### Strategies related to the CPS implementation process

A suggestion of strategy to ensure that CPS were implemented in this study was the adequacy of the type of service according to the social demands of the setting where it will be provided. Latif et al. [99] explored factors related to implementing a new community pharmacy service. Pharmacists reported the need to simplify and adapt the service to find the way that best suited the needs of local reality, since the processes involved in the implementation are complex and multifaceted. Thus, ensuring that services match the social needs of the place where it will be implemented can be a relevant strategy.

Factors related to the professionals’ profile were reported as a strategy to be performed. Elvey, Hassell e Hall [100] examine pharmacists’ perceptions of their professional identity employed in the community, hospital, and primary care sectors, both in terms of how they see themselves and how they think others view their profession. The study identified the presence of nine identities for pharmacists: the scientist, the medicine adviser, the clinical practitioner, the social care-giver, the medicine maker, the medicine supplier, the manager, the businessman, and the unremarkable character. According to the authors, many profiles can be found for a single profession. Thus, it may be a viable strategy to select profiles that are suitable for CPS in order to ensure a higher participation rate and reduce withdrawals.

##### Strengths and limitations

This study used triangulation of methods and sources in order to increase the reliability of the results. These may have helped to get a comprehensive understanding of the facilitators and strategies related to the implementation process. Moreover, this may allow the minimization of political influences in the results of the study, since the implementation process was observed from the standpoint of accredited, non-accredited pharmacists, and local health managers, who operated in different settings in the municipal healthcare network.

One of the limitations of this study was the refusal of some non-accredited pharmacists to participate in the focus groups. If more pharmacists had participated, new implementation factors could have arisen. Another limitation was that we exclusively investigated the perception of strategies of pharmacists on the implementation of CPS in healthcare systems, rather than evaluating the perception of other actors involved in the process, such as local health managers. In addition, this study did not investigate whether the strategies listed can actually bring real benefits to the implementation process.

## Conclusion

This study identified facilitators and strategies involved in CPS implementation process revealed by pharmacists and the local health manager. Facilitators to implement CPS included physical structure, human resources, training of professionals, management commitment, well-defined work processes, and interprofessional collaboration. Similarly, strategies were associated with protagonist approaches and active attitudes of pharmacists, such as reconciling logistics and clinical activities, sensitizing the healthcare team, disclosing CPS, and sensitizing and get patients by active search. In addition, this study can guide pharmacists, managers, and policy makers in the implementation of CPS in healthcare systems provided for patients, families, and communities.

## Additional file

Additional file 1: Focus group and interviews questions. (DOCX 12 kb)

## Abbreviations

COREQ: Consolidated Criteria for Reporting Qualitative Research
CPS: Clinical Pharmacy Services
