# Supplementary material for: Facilitators and strategies to implement clinical pharmacy services in a metropolis in Northeast Brazil: a qualitative approach
Source: BMC Health Serv Res. 2018 Aug 13;18:632. doi: 10.1186/s12913-018-3403-4 (PMC6090582; doi:10.1186/s12913-018-3403-4)
Supplement: Supplementary file 1 — Focus group and interviews questions. (DOCX 12 kb) [file 12913_2018_3403_MOESM1_ESM.docx]

**Questions Script**

1. What were the barriers that hampered the CPS implementation process?
2. What were the facilitators that promoted the CPS implementation process?
3. What were the strategies for implementing CPS?
